# Transplacental Foetal Blood Loss: A Common Event

**Published:** 1964-07

**Authors:** I. D. Fraser

**Affiliations:** Department of Pathology, The Bristol Royal Infirmary


### TRANSPLACENTAL FOETAL BLOOD LOSS: A COMMON EVENT

BY

I. D. FRASER, M.D.

*Department of Pathology, The Bristol Royal Infirmary*

In 1941 Levine and his colleagues postulated that the way in which Rh sensitization occurred in Rh negative mothers was by transplacental passage of Rh-positive foetal erythrocytes into the maternal circulation. At that time there were no available techniques for proving this hypothesis, and it was not known when the foetal cells entered the maternal circulation, nor how much foetal blood was required to stimulate Rh antibody formation. Weiner (1948) and Wickster (1952) both postulated that a large foetal haemorrhage into the maternal circulation could cause anaemia in the new-born baby. Chown (1954) was subsequently able to prove beyond all doubt that a large foeto-maternal transfusion could occur; after the birth of an anaemic baby, he was able to demonstrate the presence of both foetal erythrocytes (by differential agglutination) and foetal haemoglobin in the maternal blood. Furthermore he demonstrated maternal antibodies against the foetal erythrocytes 3 weeks after delivery. After Chown's observations further instances of large foeto-maternal transfusions were recorded, but the emphasis at this time appeared to be on detecting large rather than small foetal haemorrhages.

In 1957 Kleihauer, Braun and Betke evolved a method for differentiating erythrocytes containing adult haemoglobin from those containing foetal haemoglobin. Briefly they found that by fixing thin smears of normal adult and cord blood with methyl alcohol, and then treating the slides in a citric acid-phosphate buffer at pH 3·4, the denatured haemoglobin from adult erythrocytes was soluble and this left the cell, whereas denatured foetal haemoglobin was insoluble and remained within the cell. When haemoglobin stains were employed cells containing foetal haemoglobin stained up fully but cells which had contained adult haemoglobin appeared as "ghosts", only the cell outline being visible. This method, when applied to maternal blood, is extremely sensitive for detecting any foetal erythrocytes that may be present, and since its original description further instances of large foeto-maternal transfusions have appeared in the literature. Using this technique Zipursky and his colleagues in Canada (1959) examined the blood of 42 mothers within 6 days of parturition, and found small numbers of foetal cells in 11, thus indicating the occurrence of small foeto-maternal transfusions. This opened the field for more intensive observations as to the incidence of these micro-transfusions from foetus to mother. Large series from Liverpool, Bristol, Inverness, Germany and Canada have shown that when maternal blood is examined within 2 days of delivery, foetal cells can be detected in approximately 30 per cent of pregnancies where the mother and baby are ABO-compatible. If the mother and baby are ABO-incompatible the chances of detecting foetal cells within 48 hours of delivery are reduced, as the incompatible foetal cells would only be expected to survive for a few hours in the maternal circulation. The vast majority of these transfusions are very small, i.e. less than 1 ml. of foetal blood.

Most observers have confined themselves to examining post-partum blood; however some recent observations during the ante-natal period indicate that very small foeto-maternal transfusions occur in about 15 per cent of apparently normal pregnancies. The estimated volumes are very small, usually of the order of less than 0·1 ml.

This finding poses the question as to whether this is simply a normal occurrence in the ante-natal period or an indication of very small silent accidental haemorrhages. The exact time when foetal cells are most likely to enter the circulation is not quite clear, but from observations made in Bristol it seems that the highest incidence of micro-transfusion occurs during the second and third stages of labour.

In 30 per cent of normal pregnancies and labours small foeto-maternal transfusions can be expected to occur. However the chances of inducing a foeto-maternal transfusion are increased in the accidents of pregnancy and in certain obstetric procedures. In Bristol we have detected foetal cells in the maternal blood in 50 per cent of women whose pregnancies ended in abortion between the 20-24th weeks of gestation, and in 80 per cent of women who had accidental haemorrhage between the 33-36th weeks of gestation. Presumably in these abnormal cases the bigger the placenta the greater the risk of some foetal blood entering the maternal circulation. It has also been shown that the risk of inducing a placental leak is increased by Caesarean section, instrumentation, twin pregnancy, and manual removal of the placenta.

The incidence of these micro-transfusions is of course the same for both Rh-positive and the Rh-negative mothers. This must have some bearing on the possible sensitization of the Rh-negative mother if her foetus is Rh-positive. It is now almost certain that the original hypothesis of Levine is correct, as it is only the foetal erythrocyte which carries the Rh antigen, and the frequent finding of foetal cells in the blood of Rh-negative mothers shows at any rate that the latter are exposed to Rh-antigenic material.

Rh antibodies are only rarely found during the first pregnancy. Clemens and Walshe (1954) found antibodies in 0·3 per cent of mothers after one pregnancy and in 6·6 per cent after two. Nevanlinna (1953) suggested that maternal sensitization might occur in an early pregnancy, as a result of the passage of small volumes of foetal blood, but without demonstrable antibodies; and that antibodies may be detected in later pregnancies after further small antigenic stimuli. Zipursky *et al.* (1963) found that of 199 women in whom antibodies were first demonstrated during pregnancy, 67 had Rh antibodies when tested early in pregnancy and the other 132 developed antibodies later on in pregnancy. They believed that the 67 mothers with antibodies early in pregnancy had received a sufficient volume of foetal blood during a previous pregnancy to stimulate Rh antibody production, whereas the other 132, although sensitized, had required further antigenic stimuli during the ante-natal period to produce demonstrable antibodies. The fact that small volumes of foetal cells can be detected in the maternal blood during the ante-natal period in about 15 per cent of mothers adds further support to this mechanism of sensitization.

The critical volume of foetal blood required to stimulate Rh antibody production is not known but experimental work suggests that at least 1·5 ml. of Rh-positive foetal blood is required.

The technique of demonstrating foetal erythrocytes devised by the German research workers has considerably advanced our knowledge of Rh sensitization. Small foeto-maternal transfusions can be expected in about 30 per cent of normal pregnancies. Obstetricians are now well aware that careful management is required of the young Rh-negative mother married to an Rh-positive husband as everything has to be planned to avoid the risk of sensitization.

In the Haematology Department of the Royal Infirmary, Bristol, we are very keen to investigate cases of large foeto-maternal transfusions. If a practitioner is faced with an anaemic newborn baby the possibility of a large foeto-maternal transfusion should be borne in mind. It is a very simple matter to search for foetal erythrocytes in the maternal blood. All that is required is a venous anticoagulated sample from the mother, within 2 days of delivery. Samples can easily be dealt with if they are sent by post.

SUMMARY

Small foeto-maternal transfusions are a common event in obstetric practice. The risk of inducing a foetal leak is increased by certain obstetrical procedures. Recent work as to the mechanism of the sensitization during pregnancy is discussed.

### REFERENCES

Chown, B. (1954). *Lancet*, **i**, 1213.
Clemens, K. and Walshe, R. J. (1954). *Med. J. Aust.*, **ii**, 707.
Kleihauer, E., Braun, H. and Betke, K. (1957). *Klin. Wschr.*, **35**, 637.
Levine, P., Katzin, E. M. and Burnham, L. (1941). *J. Amer. med. Ass.*, **116**, 825.
Nevanlinna, H. R. (1954). *Ann. Med. exp. Fenn.*, 31, suppl. 2.
Weiner, A. S. (1948). *Amer. J. Obstet. Gynec.*, **56**, 717.
Wickster, G. Z. (1952). *ibid.* **63**, 524.
Zipursky, A., Hull, A., White, F. D. and Israels, L. G. (1959). *Lancet*, **i**, 451.
Zipursky, A., Pollock, J., Neelands, P., Chown, B. and Israels, L. G. (1963). *Lancet*, **ii**, 489.

